# Pph3 Dephosphorylation of Rad53 Is Required for Cell Recovery from MMS-Induced DNA Damage in *Candida albicans*

**DOI:** 10.1371/journal.pone.0037246

**Published:** 2012-05-14

**Authors:** Haitao Wang, Jiaxin Gao, Wanjie Li, Ada Hang-Heng Wong, Kangdi Hu, Kun Chen, Yue Wang, Jianli Sang

**Affiliations:** 1 Key Laboratory of Cell Proliferation and Regulation Biology, Ministry of Education, College of Life Sciences, Beijing Normal University, Beijing, People's Republic of China; 2 Protein Science Laboratory, School of Life Sciences, Tsinghua University, Beijing, China; 3 Institute of Molecular and Cell Biology, Agency for Science, Technology and Research (A*STAR), Singapore, Singapore; New Jersey Medical School, University of Medicine and Dentistry of New Jersey, United States of America

## Abstract

The pathogenic fungus *Candida albicans* switches from yeast growth to filamentous growth in response to genotoxic stresses, in which phosphoregulation of the checkpoint kinase Rad53 plays a crucial role. Here we report that the Pph3/Psy2 phosphatase complex, known to be involved in Rad53 dephosphorylation, is required for cellular responses to the DNA-damaging agent methyl methanesulfonate (MMS) but not the DNA replication inhibitor hydroxyurea (HU) in *C. albicans*. Deletion of either *PPH3* or *PSY2* resulted in enhanced filamentous growth during MMS treatment and continuous filamentous growth even after MMS removal. Moreover, during this growth, Rad53 remained hyperphosphorylated, MBF-regulated genes were downregulated, and hypha-specific genes were upregulated. We have also identified S461 and S545 on Rad53 as potential dephosphorylation sites of Pph3/Psy2 that are specifically involved in cellular responses to MMS. Therefore, our studies have identified a novel molecular mechanism mediating DNA damage response to MMS in *C. albicans*.

## Introduction

*Candida albicans* is a pleiomorphic fungus that can grow in three different morphological forms: budding yeast, pseudohyphae and hyphae [1], [2], rendering it an excellent model for studying cell morphogenesis [2]–[5]. The yeast-to-hyphal growth transition has been implicated in its virulence in various human superficial infections of the skin, vagina and oral epithelia [5]–[9]. Therefore, understanding the underlying mechanisms that regulate the morphological transition may provide key insights into potential strategies for developing antifungal therapeutics.

Earlier studies showed that the cAMP/protein kinase A (PKA) and mitogen-activated protein (MAP) kinase pathways play key roles in regulating the hyphal growth of *C. albicans* [7], [10], [11]. Recently, many studies revealed cell cycle checkpoints as part of alternative regulatory pathways mediating filamentous growth under various conditions that block cell cycle progression [12], [13]. For example, depletion of the G1 cyclin Cln3 [14], [15], or the mitotic cyclin Clb2 or Clb4 [16], or deletion of other cell cycle regulatory genes such as *CDC*4 or *CDC5* were shown to result in filamentous growth [17], [18]. Filamentous growth was also observed in several *C. albicans* deletion mutants defective in DNA damage repair [19], [20]. DNA damaging agents were found to cause cell cycle arrest and filamentous growth in a manner dependent on the DNA damage/replication checkpoint kinase Rad53 [21].

Rad53, the yeast homolog of human Chk2 [22], [23], is a Ser/Thr kinase that plays a pivotal role in G2/M checkpoint regulation by phosphorylating various substrates involved in cell cycle progression and/or DNA damage repair [24]–[26]. Hyperphosphorylation of Rad53 is sufficient for cell cycle arrest and its dephosphorylation leads to recovery after genotoxic stress [27]–[29]. Previous studies revealed diverse phosphorylation and dephosphorylation patterns on Rad53 under different circumstances. The phosphorylation mainly occurs in the two SQ cluster domains (SCDs). The N-terminal SCD is conserved in human Chk2, while the C-terminal SCD is unique to the yeast homologs. Several protein kinases such as Mec1, Mrc1 and Rad9 [27], [30]–[32] and phosphatases Pph3 and Ptc2 are involved in regulating Rad53 phosphorylation [28], [33], [34]. However, the sites of phosphorylation/dephosphorylation by different kinases and phosphatases and their regulation and biological significance of the phosphorylation state of particular sites remain largely unknown. It was reported that Pph3 binds to the central kinase domain of Rad53, while Ptc2 binds to its FHA1 domain, and that their deletion led to sensitivity to different genotoxic stresses [35]. However, the underlying molecular mechanisms remain elusive.

In this study, we examined the role of the phosphatase Pph3/Psy2 in regulating cellular responses to MMS and HU in *C. albicans*. We investigated how deletion of the phosphatase genes affected Rad53 phosphorylation and its ability to regulate downstream signaling and cell morphogenesis. We also obtained evidence on the potential sites for Pph3/Psy2 dephosphorylation on Rad53.

## Results

### *pph3Δ* and *psy2Δ* mutants exhibited hypersensitivity to MMS but not HU

**Figure 1:**
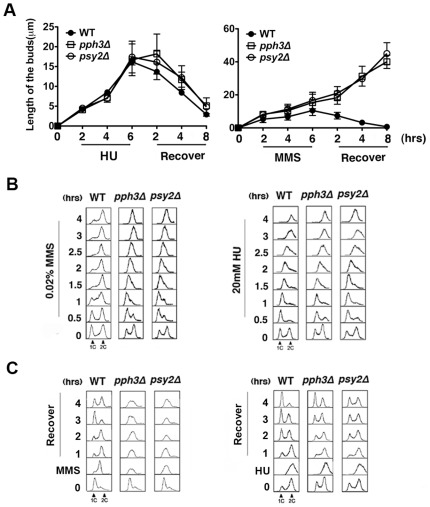
*pph3Δ* and *psy2Δ* cells exhibit pseudohyphal growth and cell cycle arrest when treated with MMS or HU. Fig 1A. Wild-type (SC5314 or BWP17), *pph3Δ* (SJL3) and *psy2Δ* (SJL6) cells were grown in liquid YPD medium supplemented with 0.02% MMS or 20 mM HU at 30°C for 6 h, washed with fresh YPD and resuspended into fresh YPD for further growth at 30°C for 8 h. Bud length was measured using ImageJ (http://rsbweb.nih.gov/ij/index.html). Each data point represents the average of 30 cells measured in 3 independent experiments. Fig 1B. The same cells as used in (A) were treated with 0.02% MMS or 20 mM HU. Cells were harvested at indicated time intervals for flow cytometry analysis. Fig 1C. The same cells as used in (A) were treated with 0.02% MMS or 20 mM HU at 30°C for 6 h and recovered in fresh YPD as described in (A). Cells were harvested at indicated time intervals for flow cytometry analysis.

Previous studies in *Saccharomyces cerevisiae* demonstrated that *PPH3* deletion led to hypersensitivity towards MMS but not HU [34]. Thus, we first determined whether the same phenomenon also occurs in *C. albicans*. Wild-type, *pph3Δ* and *psy2Δ* yeast cells (Table 1) were inoculated into liquid YPD medium containing different concentrations of MMS or HU and incubated at 30°C for 6 h, followed by recovery in fresh drug-free YPD medium for 8 h at 30°C. Microscopic examination of the genotoxin-induced cell elongation at timed intervals revealed that both *pph3Δ* and *psy2Δ* mutants exhibited cell elongation during HU treatment and returned to the yeast form of growth after drug removal in manners comparable to wild-type cells (Fig. 1A, left & Fig. S2). In comparison, during MMS treatment the mutant cells exhibited faster elongation than wild-type cells and continued to elongate throughout the entire recovery period, while the wild-type cells returned to yeast growth ∼2 h after shifting to the drug free medium (Fig. 1A, right & Fig. S1).

**Table 1:** C. albicans strains used in this study.

Strains	Relevant genotype	Source
SC5314	Wild type, clinical isolate	
BWP17	ura3/ura3 his1::hisG/his1::hisG arg4::hisG/arg4::hisG	[69]
SJL2	BWP17 pph3Δ::ARG4/pph3Δ::HIS1	[36]
SJL2.1	BWP17 pph3Δ::ARG4/pph3Δ::HIS1 PPH3:URA3	[36]
SJL3	BWP17 pph3Δ::ARG4/pph3Δ::HIS1 URA3	[36]
SJL5	BWP17 psy2Δ::ARG4/psy2Δ::HIS1	[36]
SJL5.1	BWP17 psy2Δ::ARG4/psy2Δ::HIS1 PSY2:URA3	[36]
SJL6	BWP17 psy2Δ::ARG4/psy2Δ::HIS1 URA3	[36]
SJL7	BWP17 pph3Δ::ARG4/pph3Δ::HIS1 RAD53-Myc:URA3	[36]
SJL8	BWP17 psy2Δ::ARG4/psy2Δ::HIS1 RAD53-Myc:URA3	[36]
SJL9	BWP17 RAD53-Myc-URA3	[36]
HKD1	BWP17 ptc2Δ::ARG4/ptc2Δ::HIS1	unpublished
HKD1.1	BWP17 ptc2Δ::ARG4/ptc2Δ::HIS1 URA3	unpublished
HKD2	BWP17 pph3Δ::ARG4/pph3Δ::HIS1 ptc2Δ::FRT/ptc2Δ::FRT URA3	unpublished
HT1	BWP17 RFA2-Myc-URA3	This study
HT2	BWP17 pph3Δ::ARG4/pph3Δ::HIS1 RFA2-Myc:URA3	This study
HT3	BWP17 psy2Δ::ARG4/psy2Δ::HIS1 RFA2-Myc:URA3	This study
HT4	BWP17 ptc2Δ::ARG4/ptc2Δ::HIS1 RFA2-Myc:URA3	This study
HT5	BWP17 pph3Δ::ARG4/pph3Δ::HIS1 ptc2Δ::FRT/ptc2Δ::FRT RFA2-Myc:URA3	This study
WY3	rad53Δ::ARG4/rad53Δ::URA3	[19]
HT6	rad53Δ::ARG4/rad53Δ::URA3 RAD53:HIS1	This study
HT7	rad53Δ::ARG4/rad53Δ::URA3 rad53T327A:HIS1	This study
HT8	rad53Δ::ARG4/rad53Δ::URA3 rad53T327D:HIS1	This study
HT9	rad53Δ::ARG4/rad53Δ::URA3 rad53S350A:HIS1	This study
HT10	rad53Δ::ARG4/rad53Δ::URA3 rad53S350D:HIS1	This study
HT11	rad53Δ::ARG4/rad53Δ::URA3 rad53S351A:HIS1	This study
HT12	rad53Δ::ARG4/rad53Δ::URA3 rad53S351D:HIS1	This study
HT13	rad53Δ::ARG4/rad53Δ::URA3 rad53S461A:HIS1	This study
HT13.1	rad53Δ::ARG4/rad53Δ::URA3 rad53S461A-Myc:HIS1	This study
HT14	rad53Δ::ARG4/rad53Δ::URA3 rad53S461D:HIS1	This study
HT14.1	rad53Δ::ARG4/rad53Δ::URA3 rad53S461D-Myc:HIS1	This study
HT15	rad53Δ::ARG4/rad53Δ::URA3 rad53S545A:HIS1	This study
HT15.1	rad53Δ::ARG4/rad53Δ::URA3 rad53S545A-Myc:HIS1	This study
HT16	rad53Δ::ARG4/rad53Δ::URA3 rad53S545D:HIS1	This study
HT16.1	rad53Δ::ARG4/rad53Δ::URA3 rad53S545D-Myc:HIS1	This study
HT17	rad53Δ::ARG4/rad53Δ::URA3 rad53S695A:HIS1	This study
HT18	rad53Δ::ARG4/rad53Δ::URA3 rad53S695D:HIS1	This study
HT19	rad53Δ::ARG4/rad53Δ::URA3 rad53S455A/S457A/S459A/S461A:HIS1	This study
HT20	rad53Δ::ARG4/rad53Δ::URA3 rad53S455D/S457D/S459D/S461D:HIS1	This study
HT21	rad53Δ::ARG4/rad53Δ::FRT rad53S461A:HIS1 RFA2-Myc:URA3	This study
HT22	rad53Δ::ARG4/rad53Δ::FRT rad53S461D:HIS1 RFA2-Myc:URA3	This study
HT23	rad53Δ::ARG4/rad53Δ::FRT rad53S545A:HIS1 RFA2-Myc:URA3	This study
HT24	rad53Δ::ARG4/rad53Δ::FRT rad53S545D:HIS1 RFA2-Myc:URA3	This study

Flow cytometry analysis showed that wild-type cells were arrested with a 2C DNA content after 2 h MMS treatment, but *pph3Δ* and *psy2Δ* mutants appeared to progress slowly through or arrested in S phase (Fig. 1B, left). In comparison, all three strains responded similarly to HU treatment, exhibiting a slow progression through S phase (Fig. 1B, right). In addition, both the *pph3Δ* and *psy2Δ* mutants reentered the cell cycle 4 h after HU removal (Fig. 1C, right), but remained arrested in S phase even 4 h after MMS removal (Fig. 1C, left).

Furthermore, wild-type cells adapted to MMS after several hours as reported for *S. cerevisiae* [37], [38]. Even in the continuous presence of the genotoxin, wild-type cells were able to exit from the DNA damage-induced cell cycle arrest, re-enter the cell cycle, and switch from filamentous growth back to yeast growth (Fig, S1). In contrast, *pph3*Δ and *psy2*Δ cells remained arrested and continued to elongate for a long period of time before finally losing viability. This phenomenon was not observed with HU treatment (Fig. S2). Hence, we conclude that Pph3/Psy2 has a role in regulating cellular response to MMS but not HU in *C. albicans.*

### Deletion of *PPH3* and *PSY2* resulted in Rad53 hyperphosphorylation

**Figure 2:**
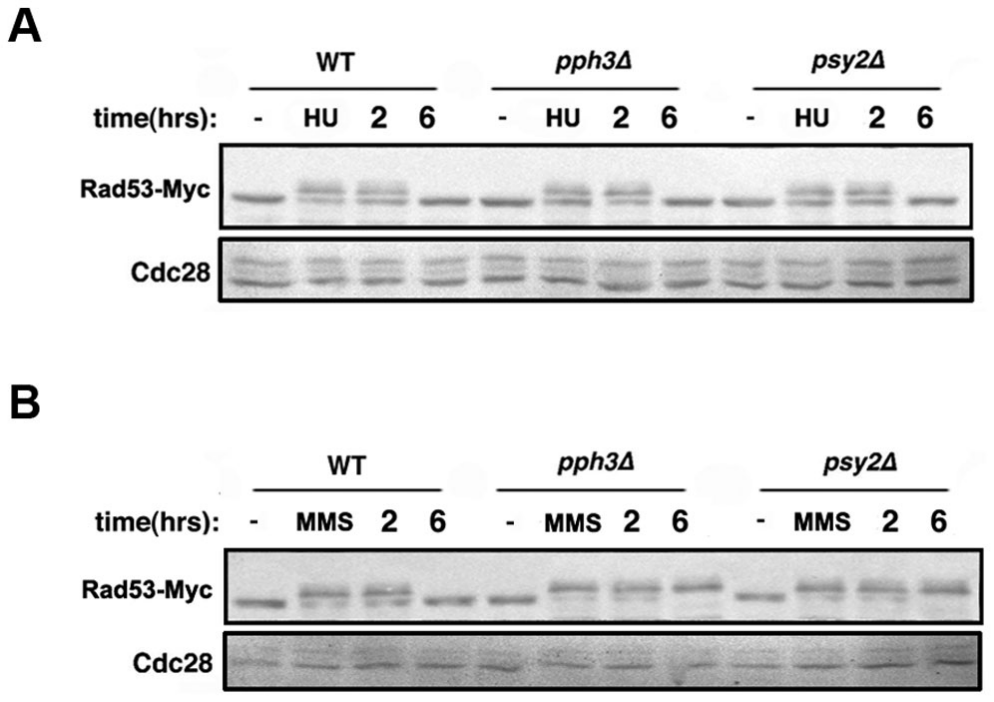
Rad53 undergoes hyperphosphorylation in response to HU and MMS. Fig 2A. Rad53 hyperphosphorylation in HU-treated cells. SJL9 (wild type with *RAD53-Myc*), SJL7 (*pph3Δ RAD53-Myc*), and SJL8 (*psy2Δ RAD53-Myc*) cells were incubated at 30°C in YPD containing 20 mM HU for 4 h. Cells were then washed and recovered with fresh YPD for the indicated times. Whole cell lysates were used for immunoblot analysis with anti-Myc antibody. Untreated cells were used as control. Cdc28 was probed with anti-PSTAIRE antibody as loading control. Fig 2B. Rad53 hyperphosphorylation in MMS-treated cells. SJL9 (wild type with *RAD53-Myc*), SJL7 (*pph3Δ RAD53-Myc*), and SJL8 (*psy2Δ RAD53-Myc*) cells were incubated at 30°C in YPD containing 0.02% MMS for 4 h. Cells were then washed and recovered with fresh YPD for the indicated times. Whole cell lysates were used for immunoblot analysis with anti-Myc antibody. Untreated cells were used as control. Cdc28 was probed with anti-PSTAIRE antibody as loading control.

Rad53 was shown to be a substrate of the Pph3/Psy2 complex in both *S. cerevisiae* and *C. albicans* [34], [38]. Furthermore, Rad53 hyperphosphorylation was shown to cause cell cycle arrest and filamentous growth in *C. albicans* [38]. Next, we examined the phosphorylation state of Rad53 in response to MMS and HU treatment. Western blotting of C-terminally Myc-tagged Rad53 demonstrated that in wild-type cells Rad53 was hyperphosphorylated after both MMS (Fig. S3B) and HU treatment (Fig. S3A), and became dephosphorylated 6 h after HU or MMS removal (Fig. 2A&B). Both *pph3Δ* and *psy2Δ* mutants also showed a similar course of Rad53 dephosphorylation after HU removal (Fig. 2A). However, Rad53 hyperphosphorylation persisted in *pph3Δ* and *psy2Δ* mutants even at 6 h after MMS removal (Fig. 2B), indicating that Rad53 dephosphorylation during recovery from MMS treatment is dependent on the Pph3/Psy2 complex.

### Deletion of *PPH3* and *PSY2* resulted in dysregulated gene expression

Next, we investigated the physiological significance of Rad53 hyperphosphorylation. Swi6p was demonstrated in *S. cerevisiae* to be phosphorylated by Rad53 to trigger G1 arrest by inhibiting the transcriptional activity of MBF [39], [40]. MBF is the Mbp1/Swi6 transcriptional complex that regulates the expression of various genes related to cell cycle progression. Therefore, we examined the expression of MBF-regulated genes [40]–[42] by RT-PCR, qPCR and Northern blot analysis in *C. albicans* during MMS treatment and the recovery from it.

**Figure 3:**
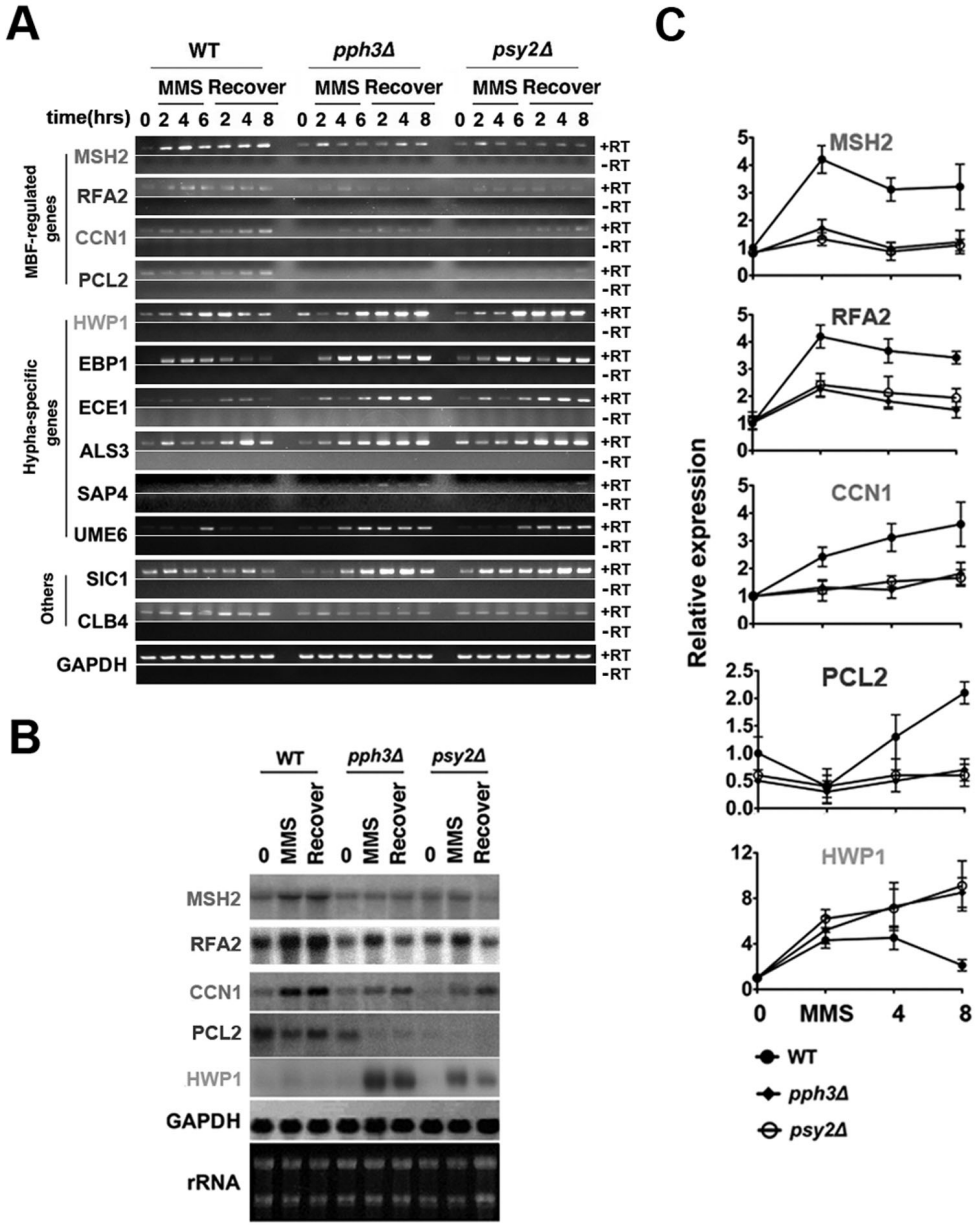
Detection of Rad53 downstream signalling by RT-PCR, Northern blot and qPCR in *pph3Δ* and *psy2Δ* cells. Fig 3A. Wild-type (S5314 or BWP17), *pph3Δ* (SJL3) and *psy2Δ* (SJL6) cells were incubated at 30°C in YPD containing 0.02% MMS and then recovered with fresh YPD over the indicated time period. RNA extracted from harvested cells at indicated time points was used for RT-PCR analysis. PCR amplifications in absence of retrotranscriptase for each sample was used as negative controls in RT-PCR. GAPDH was used as loading control. Fig 3B. Wild-type (S5314 or BWP17), *pph3Δ* (SJL3) and *psy2Δ* (SJL6) cells were incubated at 30°C in YPD containing 0.02% MMS for 6 h and then recovered with fresh YPD for 6 h. RNA was extracted and subject to Northern blot analysis. GAPDH was used as control and rRNA was shown to indicate RNA integrity. Fig 3C. Wild-type (S5314 or BWP17), *pph3Δ* (SJL3) and *psy2Δ* (SJL6) cells were incubated at 30°C in YPD containing 0.02% MMS for 6 h and then recovered with fresh YPD over the indicated time period. RNA was extracted and reverse transcribed into cDNA at the indicated time points for qPCR analysis. All data represent the mean of 3 independent experiments.

Results showed that there is an overall downregulation of MBF-regulated genes, such as *MSH2*, *RFA2*, *CCN1* and *PCL2*, upon MMS treatment and during recovery in the *pph3Δ* or *psy2Δ* mutant as compared to wild-type cells (Fig. 3A&B). Upon MMS treatment, upregulation of *MSH2*, *RFA2* and *CCN1* was observed in wild-type, but to a minor level in the *pph3Δ* and *psy2Δ* mutant; while *PCL2* was downregulated in all three strains (Fig. 3A–C). Higher than normal levels of *MSH2*, *CCN1* and *RFA2* persisted in wild-type cells after recovery, while *RFA2* returned to normal levels in the *pph3Δ* and *psy2Δ* mutant during recovery (Fig. 3B&C). In contrast, *PCL2* exhibited higher-than-normal expression levels after MMS recovery in wild-type cells, but exhibited near normal expression levels after recovery in the *pph3Δ* and *psy2Δ* mutant (Fig. 3B&C). Negative controls without reverse transcriptase was used in the RT-PCR experiments to rule out genomic DNA contamination during PCR amplification (Fig. 3A). GADPH was used as loading control, and rRNA was included to indicate RNA integrity in Northern blot experiment (Fig. 3B).

Because filamentous growth was observed after MMS treatment, we also examined the expression of several known hypha-specific genes including *HWP1*, *EBP1*, *ECE1*, *ALS3*, *SAP4* and *UME6*. Interestingly, expression of hypha-specific genes was significantly higher in *pph3Δ* and *psy2Δ* cells than in wild-type cells during MMS recovery (Fig. 3A&B). Hence, this phenomenon is consistent with the observed cell elongation in *C. albicans* upon MMS treatment (Fig. S1). In addition to hypha-specific genes, we also monitored the cyclin gene *CLB4* and its inhibitor *SIC1*. *CLB4* showed sustained downregulation in both the *pph3Δ* and *psy2Δ* mutant as compared to wild type cells. In contrast, expression of its inhibitor gene *SIC1* was higher in *pph3Δ* and *psy2Δ* cells than in wild-type cells under normal conditions and increased further after MMS treatment (Fig. 3A), supporting the observation that *pph3Δ* and *psy2Δ* cells were unable to recover from cell cycle arrest. However, whether these phenomena are directly related to Rad53 remains to be investigated.

**Figure 4:**
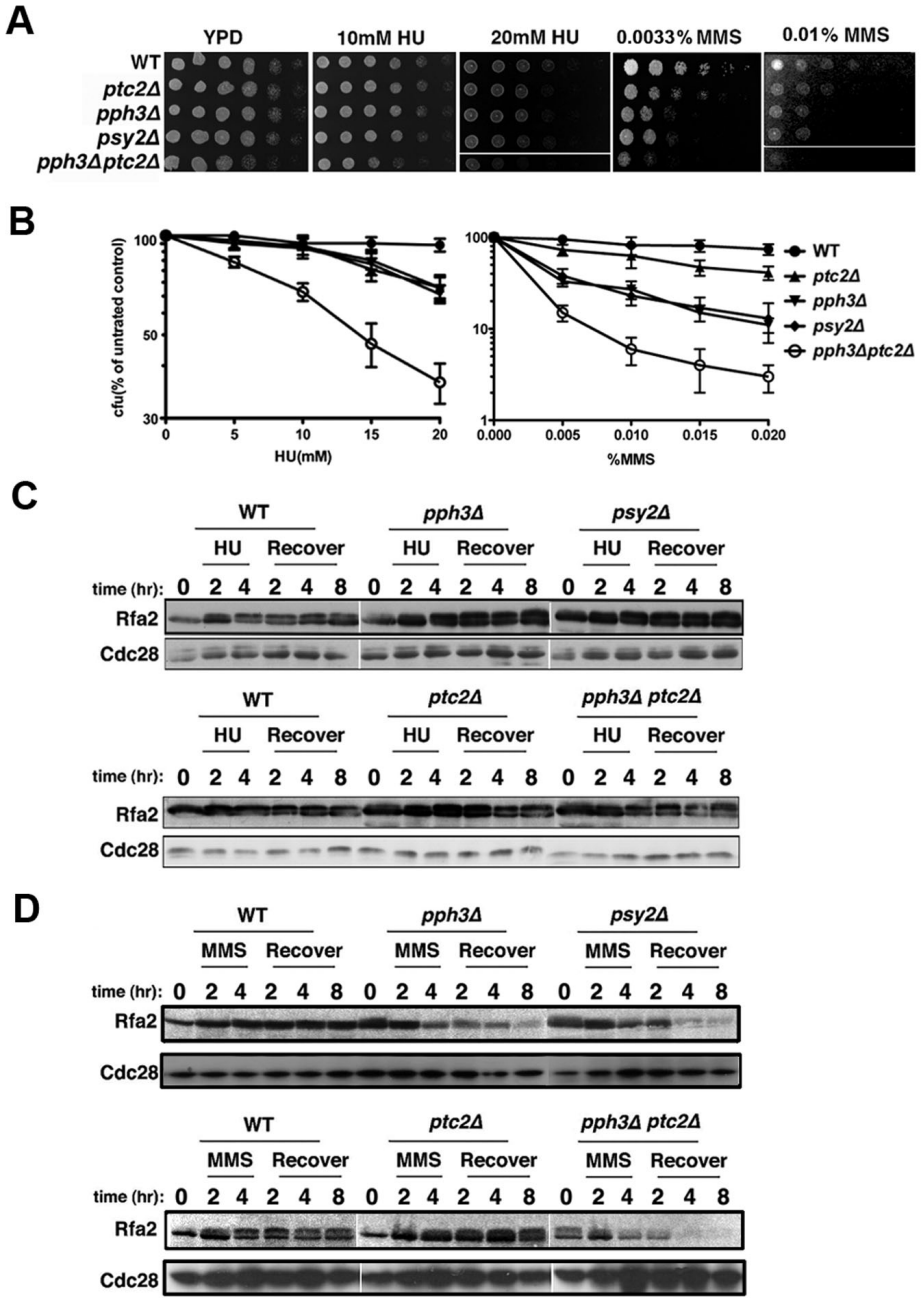
Rfa2 was downregulated in *pph3Δ* and *pph3Δ ptc2Δ* mutant upon MMS but not HU treatment. Fig 4A. Wild-type (HT1), *pph3Δ* (HT2), *psy2Δ* (HT3), *ptc2Δ* (HT4) and *pph3Δ ptc2Δ* (HT5) cells were 10-fold serially diluted, spotted onto YPD plates containing different concentrations of HU or MMS, and incubated at 30°C for 24 h. Fig 4B. Approximately equal numbers of yeast cells were spread onto YPD plates containing different concentrations of HU and MMS for incubation at 30°C for 2 d. Percentage of viability was expressed as colony-forming units (CFU) of HU- or MMS- treated mutants compared to untreated wild-type control. Fig 4C. Wild-type (HT1), *pph3Δ* (HT2), *psy2Δ* (HT3), *ptc2Δ* (HT4) and *pph3Δ ptc2Δ* (HT5) cells expressing C-terminally Myc-tagged Rfa2 were incubated at 30°C in YPD containing 20 mM HU and recovered with fresh YPD over the indicated time period. Untreated cells was used as control. Total protein was extracted from harvested cells at the indicated time points and subject to immunoblot analysis with anti-Myc antibody. Cdc28 was probed with anti-PSTAIRE antibody as loading control. Fig 4D. Wild-type (HT1), *pph3Δ* (HT2), *psy2Δ* (HT3), *ptc2Δ* (HT4) and *pph3Δ ptc2Δ* (HT5) cells expressing C-terminally Myc-tagged Rfa2 were incubated at 30°C in YPD containing 0.02% MMS and recovered with fresh YPD over the indicated time period. Untreated cells were used as control. Total protein was extracted from harvested cells at the indicated time points and subject to immunoblot analysis with anti-Myc antibody. Cdc28 was probed with anti-PSTAIRE antibody as loading control.

Northern blot (Fig. 3C) and qPCR (Fig. 4D) analyses produced consistent results in the expression levels of *MSH2*, *RFA2*, *CCN1*, *PCL2* and *HWP1*, which are genes involved in either cell cycle regulation or hyphal growth. Among these genes, downregulation of *RFA2* in *pph3Δ* and *psy2Δ* mutants after MMS recovery was further confirmed by Western blot analyses (Fig. 4D). Therefore, our results suggest that Rad53 hyperphosphorylation resulting from *PPH3* and *PSY2* deletion triggers its downstream signaling, which may contribute to MMS sensitivity.

Pph3 and Ptc2 are two phosphatases that have been implicated in Rad53 dephosphorylation during genotoxic stress [28], [33], [34]. Next, we asked whether *RFA2* downregulation during MMS treatment is specific to *PPH3* deletion. To this end, we spotted BWP17, *pph3Δ*, *psy2Δ, ptc2Δ* and *pph3Δ ptc2Δ* cells onto YPD plates containing different concentrations of MMS or HU, and incubated at 30°C for 24 h. Results showed that in the absence of genotoxin, the *pph3Δ*, *psy2Δ* and *ptc2Δ* mutant exhibited normal growth indistinguishable from the wild-type strain; however, the *pph3Δ ptc2Δ* mutant grew more slowly (Fig. 4A), suggesting some degree of functional redundancy between Pph3 and Ptc2 in cell growth. In the presence of genotoxins, while all mutants showed increased sensitivity, the *pph3Δ ptc2Δ* mutant was the most sensitive (Fig. 4A&B), indicating that Pph3 and Ptc2 have both independent and redundant functions important for cell viability in response to genotoxic stress. Ptc2 seemed to play a lesser role in MMS sensitivity than Pph3, because the *ptc2Δ* mutant displayed lower MMS sensitivity than both the *pph3Δ* and *psy2Δ* mutant (Fig. 4A&B). All the single-gene deletion mutants exhibited similar sensitivity to HU (Fig. 4A&B). Western blotting of Rfa2-Myc in *pph3Δ*, *psy2Δ*, *ptc2Δ* and *pph3Δ ptc2Δ* mutants showed that Rfa2 cellular levels were comparable in HU-treated mutant cells (Fig. 4C). In comparison, its cellular level diminished significantly after MMS treatment in *pph3Δ*, *psy2Δ* and *pph3Δ ptc2Δ* mutants, while remained unaffected in wild-type and *ptc2Δ* cells (Fig. 4D). Therefore, these results suggest that downregulation of Rfa2 is closely associated with Pph3 dephosphorylation of Rad53.

Double bands were observed of Rfa2 after HU and MMS treatment (Fig. 4C&D). Previous studies demonstrated that RPA2, the human homolog of Rfa2, is hyperphosphorylated under various genotoxic stresses [43]–[48]. Thus, the upper band may represent phosphorylated Rfa2. However, we have not studied the post-translational modification of Rfa2. Nevertheless, this should not compromise our conclusion that Rfa2 downregulation is a result of *PPH3* deletion.

### Identification of potential MMS-related dephosphorylation sites on Rad53

**Figure 5:**
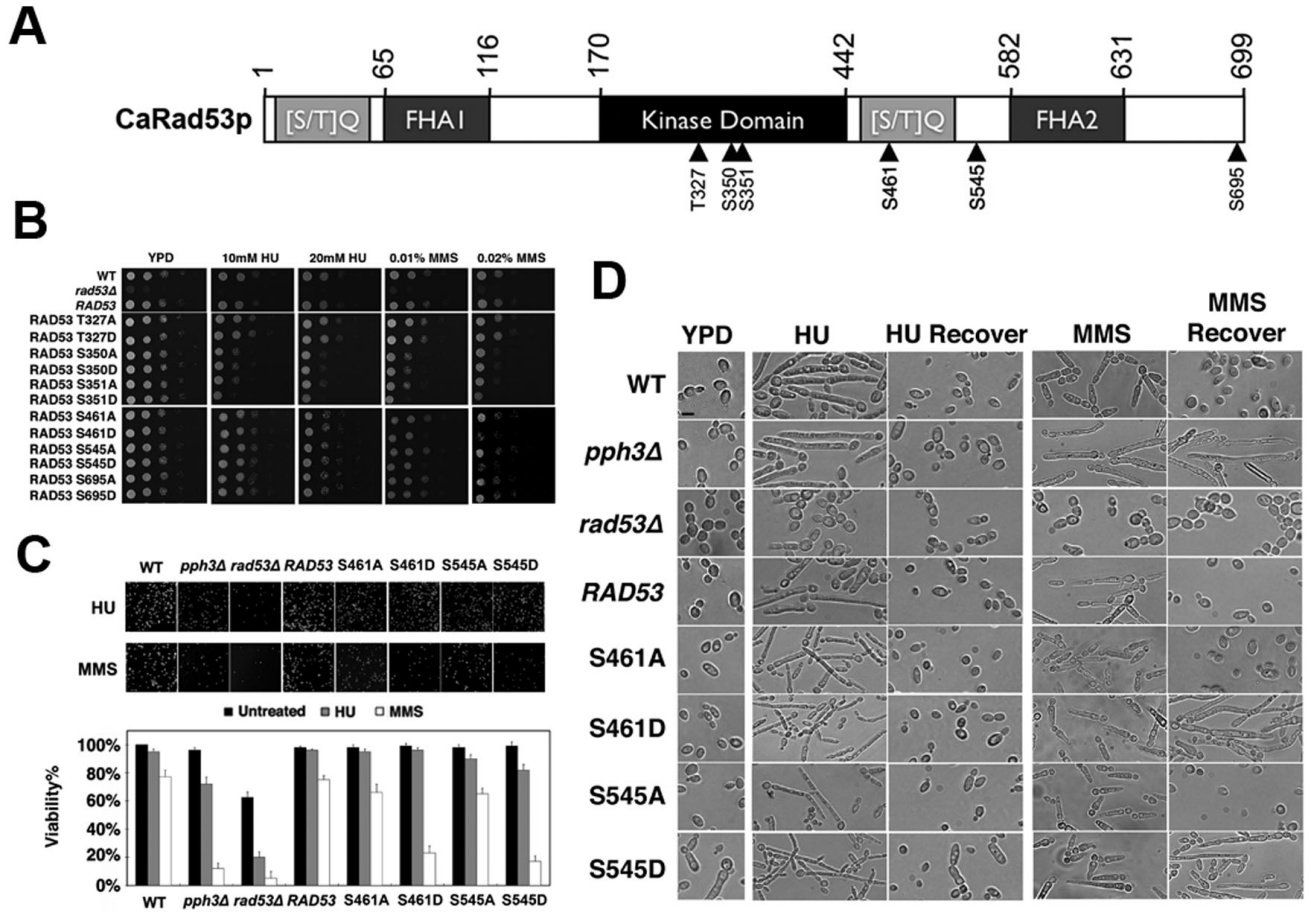
Viability assays of Rad53 phosphomimic mutants upon MMS and HU treatment. Fig 5A. Domain organizations of *C. albicans* Rad53 and *S. cerevisiae* Rad53. Arrowheads mark [S/T]Q amino acid mutant site. Amino acids at domain boundaries are indicated by numbers. Schematic description of the strategy for integrating *RAD53* wild-type and mutant alleles at the *RAD53* chromosomal locus (for details, see *Materials and Methods*.). Fig 5B. Cells of wild-type (S5314 or BWP17), *rad53Δ* (WY3), the rescued *RAD53* (HT6) and the various strains expressing mutant alleles of *RAD53* (HT7–18 refer to Table 1) were 10-fold serially diluted, spotted onto YPD plates containing different concentrations of HU or MMS, and incubated at 30°C for 24 h. Fig 5C. Approximately equal numbers of cells of wild type (S5314 or BWP17), *pph3Δ* (SJL3), *rad53Δ* (WY3), the rescued *RAD53* (HT6) and the various strains expressing mutant alleles of *RAD53* (HT13–16 *rad53-S461A*, *rad53-S461D*, *rad53-S545A*, *rad53-S545D*) were treated with 20 mM HU or 0.02 mM for 2 h in liquid culture and then spread onto YPD plates for incubation at 30°C for 2 d. Percentage of viability was expressed as CFU of the untreated mutants compared to untreated wild-type cells, and CFU of HU-treated or MMS-treated mutants was compared to their untreated counterpart. All data show the average of three independent experiments with error bars. Fig 5D. Cells of wild type (S5314 or BWP17), *pph3Δ* (SJL3), *rad53Δ* (WY3), the rescued *RAD53* (HT6) and the various strains expressing mutant alleles of *RAD53* (HT13–16 *rad53-S461A*, *rad53-S461D*, *rad53-S545A*, *rad53-S545D*) were grown in liquid YPD medium supplemented with 0.02% MMS or 20 mM HU at 30°C for 4 h. Cells were collected for microscopic examination. 0.02% MMS or 20 mM HU treated cells washed with fresh YPD and recovered into MMS-free and HU-free YPD at 30°C for 8 h. Cells were collected for microscopic examination. (Bar = 5 µm)

To identify potential phosphorylation sites on Rad53 that may be responsible for MMS sensitivity, we performed phosphomimetic mutagenesis on previously reported phosphorylation sites (Fig. 5A). Results showed that the Rad53 phosphomimetic mutants of S461D and S545D (corresponding to 489 and 560 in *S. cerevisiae*, GeneID 855950) exhibited higher sensitivity to MMS but not HU than the wild-type strain (Fig. 5B). Viability of these mutants also dropped dramatically after MMS treatment, but they were able to recover from HU treatment (Fig. 5C, Table 2). Moreover, the two mutants remained in pseudohyphal form 10 h after MMS withdrawal, but could fully return to the yeast growth after HU treatment (Fig. 5D, Table 2).

**Table 2:** Effects of the Rad53 S/T mutations on DNA checkpoint-mediated function.

	Elongated cell^a^ (%)		Elongated cell after recovery^a^ (%)		Recovery rate^b^	
	HU	MMS	HU	MMS	HU	MMS
WT	95	83	4	10	+++++	+++++
*pph3Δ*	97	98	12	99*	++++	+*
*rad53Δ*	2	1	1	1	+	+
RAD53	93	82	3	12	+++++	+++++
*rad53*-T327A	92	85	9	11	+++++	+++++
*rad53*-S350A	91	88	5	8	+++++	++++
*rad53*-S351A	93	90	9	16	+++++	++++
*rad53*-S461A	90	92	5	9	+++++	+++++
*rad53*-S545A	93	91	8	12	+++++	+++++
*rad53*-S695A	93	89	21	23	+++++	+++++
*rad53*-S4A**^c^**	95	94	8	15	+++++	+++++
*rad53*-T327D	93	88	8	18	+++++	++++
*rad53*-S350D	93	90	9	15	+++++	++++
*rad53*-S351D	91	89	14	40	++++	+++
*rad53*-S461D	93	95	10	93*	+++++	++**
*rad53*-S545D	98	99	32	90*	+++++	++**
*rad53*-S695D	91	94	18	22	+++++	+++++
*rad53*-S4D**^c^**	93	95	14	95*	+++++	++**

a) Stationary-phase yeast cells were treated with 20 mM HU or 0.02 mM MMS in fresh YPD for 6 h, recover in fresh YPD for 8 h and the fractions of cells with an elongated bud (length of bud 1.5 times that of the mother) were counted.

b) Equivalent numbers of yeast cells were treated with 20 mM HU or 0.02 mM MMS for 2 h and then same cell diluted before being spread onto YPD plates for counting colony-forming units from few (+) to many (+++++) after a 2-d incubation at 30°C. Indicates * P value<0.01; ** *P* value<0.05 compared with values from the control WT cells.

c) *rad53*-S4A mean a quadruple mutant of S455A/S457A/S459A/S461A; *rad53*-S4D mean a a quadruple mutant of S455D/S457D/S459D/S461D.

On the other hand, the Rad53-S351D mutant (corresponding to S375 in *S. cerevisiae* Rad53p) exhibited higher sensitivity to both HU and MMS as compared to wild-type and the Rad53-S461D and Rad53-S545D mutants (Fig. 5B). However, it could partially recover from both HU and MMS treatment (Table 2), suggesting that S351 is less important in determining MMS sensitivity than S461 and S545. Furthermore, the stronger phenotype of the S to D mutation at the residues of S351, S461 and S545 suggests that phosphorylation at these sites potentially affects kinase function. Therefore, we conclude that phosphorylation at S461 and S545 appears to have a more important role in determining MMS sensitivity.

**Figure 6:**
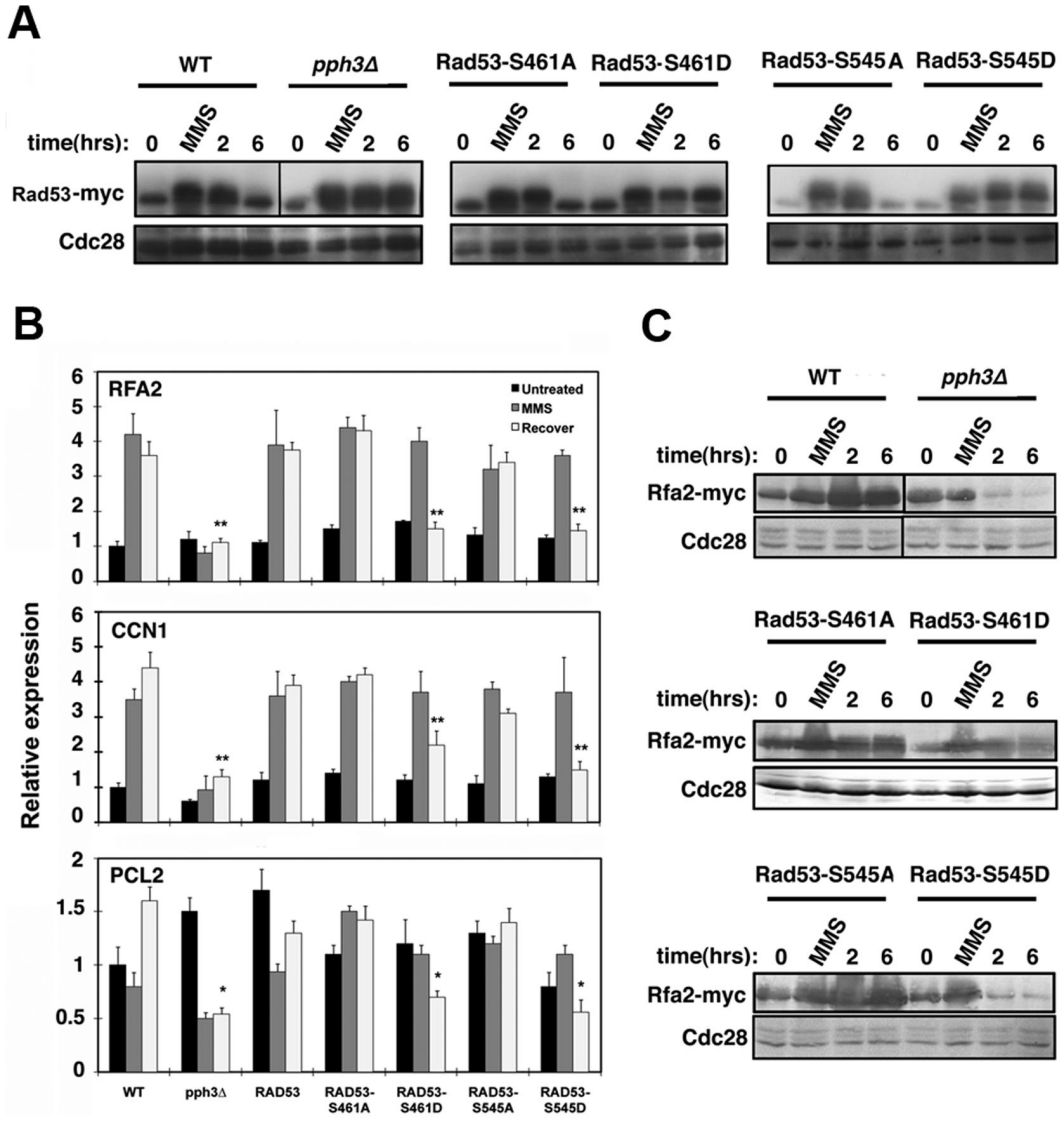
Detection of Rad53 downstream signaling by qPCR and Western blotting in *pph3Δ* cells and cells expressing various mutant alleles of *RAD53* upon MMS treatment. Fig 6A. Wild-type (SJL9), *pph3Δ* (SJL7), and the various strains expressing C-terminally Myc-tagged mutant alleles of *RAD53* (HT13.1–16.1 *rad53-S461A*, *rad53-S461D*, *rad53-S545A*, *rad53-S545D* cells were incubated at 30°C in YPD containing 0.02% MMS for 4 h, then washed and recovered with fresh YPD for the indicated times. Whole cell lysates were used for immunoblot analysis with anti-Myc antibody. Untreated cells were used as control. Cdc28 was probed with anti-PSTAIRE antibody as loading control. Fig 6B. Cells of wild type (S5314 or BWP17), *pph3Δ* (SJL3), *rad53Δ* (WY3), the rescued *RAD53* (HT6) and the various strains expressing mutant alleles of *RAD53* (HT13–16 *rad53-S461A*, *rad53-S461D*, *rad53-S545A*, *rad53-S545D*) were were incubated at 30°C in YPD containing 20 mM HU and 0.02% MMS for 4 h, and then recovered with fresh YPD for 6 h. RNA was extracted and reverse transcibed into cDNA for qPCR analysis. All data represent the mean of 3 independent experiments. (* P value<0.01; ** *P* value<0.05). Fig 6C. Cells of wild type (HT1), *pph3*Δ (HT2) and the various *RAD53* mutants expressing C-terminally Myc-tagged Rfa2 (HT21–24 *rad53-S461A Rfa2-myc, rad53-S461D Rfa2-myc*, *rad53-S545A Rfa2-myc* and *rad53-S545D Rfa2-myc*) were incubated at 30°C in YPD containing 0.02% MMS for 4 h and recovered with fresh YPD over the indicated time period. Untreated cells were used as control. Total protein was extracted from harvested cells at the indicated time points and subject to immunoblot analysis with anti-Myc antibody. Cdc28 was probed with anti-PSTAIRE antibody as loading control.

To gain evidence that phosphorylation at the above residues on Rad53 is biologically relevant, we first confirmed that the S to D mutants resulted in sustained Rad53 hyperphosphorylation after MMS treatment and recovery (Fig. 6A) but not HU (Fig. S4). Next, we used qPCR to examine the expression of Rad53 downstream genes after MMS treatment and recovery. qPCR results showed that *RFA2* as well as the cyclin genes *of CCN1* and *PCL2* were downregulated in both Rad53-S461D and Rad53-S545D mutants after MMS treatment and during the recovery, while there was no obvious difference between the Rad53-S461A and Rad53-S545A mutants and the wild type (Fig. 6B). Western blotting analysis revealed that Rfa2 levels were not recovered in the Rad53-S461D and Rad53-S545D mutants, similar to results obtained in the *pph3Δ* mutant (Fig. 6C). Hence, our results strongly suggest that failure of dephosphorylation at S461 and S545 on Rad53 may be responsible for the MMS-induced pseudohyphal growth and sensitivity in *pph3Δ* and *psy2Δ* mutants.

## Discussion

We have previously shown that Pph3 and its regulatory subunit Psy2 are required for recovery from MMS and cisplatin treatment in *C. albicans* [38]. Here, we demonstrated that *pph3*Δ and *psy2*Δ mutants exhibited hypersensitivity to MMS but not HU, consistent with earlier discoveries in *S. cerevisiae* [34]. Moreover, our results demonstrate that such hypersensitivity is closely related to CaRad53 hyperphosphorylation and that Pph3/Psy2 plays a pivotal role. Consistent with our results, Bazzi, et al. [49] showed that Glc7 but not Pph3 promoted disappearance of hyperphosphorylated Rad53 and cell's recovery after HU treatment.

Hyperphosphosphorylation of CaRad53 was observed in our *pph3*Δ and *psy2*Δ mutants upon MMS treatment. Rad53 is the yeast homolog of the mammalian tumor suppressor Chk2. It is an important checkpoint kinase that is activated in response to genotoxic stress and deactivated upon stress removal in order to exit from cell cycle arrest [23]. Rad53 deactivation is achieved through its dephosphorylation by various phosphatatases including the human PP4 homolog of Pph3, the human PP2C homologs of Ptc2 and Ptc3 [28], and the human PP1 homolog of Glc7 [49]. A large body of evidence demonstrated that different phosphorylation patterns prevail on Rad53 under different genotoxic stresses. This is regulated by different activities of various kinases and phosphatases, some of which may be redundant. For instance, *pph3*Δ cells are hypersensitive to phleomycin, while *ptc2*Δ, *ptc3*Δ and *ptc2*Δ *ptc3*Δ cells are not; on the contrary, *ptc2*Δ *ptc3*Δ cells are hypersensitive to 4-NQO while *pph3*Δ mutants are not [35]. Alternatively, Glc7, but not Ptc2, Ptc3 or Pph3, is required for recovery from an HU-induced checkpoint, while it is dispensable for checkpoint inactivation during MMS exposure. Here, we observed that the *pph3*Δ *ptc2*Δ double KO mutant exhibited higher sensitivity to MMS than the *pph3*Δ mutant, suggesting a possible role for Ptc2 in MMS sensitivity in *C. albicans*. Hence, a consensus is that different signaling pathways are activated in response to different genotoxic stresses, leading to different phosphorylation patterns of Rad53 and activation of different downstream signaling pathways.

Swi6 is a substrate of Rad53 and controls the G1/S cell cycle checkpoint. It interacts with Swi4 in the SBF complex associates with Mbp1 in the MBF complex in *S. cerevisiae* [50]–[54]. The SBF/MBF complexes are regulated through phosphorylation of Swi6 by Rad53. In *S. cerevisiae,* Rad53-dependent phosphorylation of Swi6 delayed the transition to S phase, possibly by inhibiting *CLN* transcription [46]. In *C. albicans*, cells lacking Swi4 and Swi6 demonstrated pronounced downregulation of the G1 cyclin genes *CCN1* and *PCL2* [55]. We observed a similar scenario of downregulation of MBF-regulated genes in the *pph3*Δ mutant upon MMS treatment. In addition, *RFA2* downregulation was only observed in the *pph3*Δ mutant but not in the *ptc2Δ* mutant upon MMS treatment, providing evidence for a Pph3-mediated dephosphorylation event specific for regulating the transcription of one, if not all, Swi6-regulated genes. Therefore, we deduce that such phenomenon results from the dysregulation of Pph3-dependent dephosphorylation of CaRad53.

Furthermore, downregulation of Rfa2 might also contribute to the pseudohyphal phenotype in the *pph3*Δ mutant upon MMS treatment in addition to the downregulation of SBF/MBF genes. Rfa2 is a conserved single strand DNA (ssDNA) binding protein that forms a heterotrimeric complex with two other subunits Rfa1 and Rfa3 to stabilize ssDNA during DNA replication, repair and recombination [56], [57]. *RFA2* mutations were demonstrated to induce S phase arrest in *S. cerevisiae* [58]. Here, we demonstrated that Rfa2 was downregulated upon Rad53 hyperphosphorylation in *pph3*Δ mutant. Thus, it is likely that changes in Rfa2 cellular levels contribute to the hyphal growth of *C. albicans* in addition to the downregulation of SBF/MBF genes.

Numerous studies have investigated the phosphorylation status of Rad53 under different genotoxic stresses [32], [59]–[62]. Different phosphorylation sites on Rad53 have been mapped by mass spectrometry analysis after MMS, HU and 4-NQO treatment [32], [59]. Based on these results together with sequence alignment, we mutated Thr327, Ser350, Ser351, Ser461, Ser545 and Ser695 in *C. albicans* Rad53 which correspond to Ser350, Ser373, Ser375, Ser489, Ser560 and Ser747 in *S. cerevisiae* Rad53 respectively. We observed that the S461D and S545D single amino-acid mutants (Figure 5, Table 2), and the S455D/S457D/S459D/S461D quadruple mutant (Table 2), which are located in the C-terminal SCD (SQ Cluster Domain), displayed stronger phenotypes than their S to A counterparts upon MMS treatment. Sweeney [63] provided evidence that a truncated fragment of ScRad53 (aa170–512) containing S485E/S489E mutations (corresponding to CaRad53-S457E/S461E) elevated ScRad53 trans-autophosphorylation activity *in vitro*. These data suggest that the C-terminal SCD might confer MMS hypersensitivity in *C. albicans* via trans-autophosphorylation of CaRad53. In support of this hypothesis, we observed that strains expressing the CaRad53-S461D and CaRad53-S545D proteins became hyperphosphorylated after MMS treatment *in vivo* but behaved similarly as the wild-type protein in unperturbed conditions (Fig. 6A). Thus, we believe that CaRad53 S461 and S545 contribute to but are not fully responsible for activating the kinase activity. Additional factors and/or pathways must be involved which need to be further investigated.

Earlier studies demonstrated that Psy2 binds to the kinase domain of Rad53 in *S. cerevisiae* [34]. Based on published mass spectrometry results [32], [59]–[63], Ser351 is phosphorylated upon MMS treatment. Therefore, we performed viability assays using the CaRad53-S351 mutants. Both S351D and S351A mutants exhibited lower viability upon MMS treatment, with the S to D mutant exhibiting a slightly stronger phenotype. We thus deduce that this mutation might have altered the kinase activity due to its location in the activation loop of the Rad53 kinase. Hence, we propose that S461 and S545 have a more important role in determining MMS sensitivity in *C. albicans*.

Sequence alignment of ScRad53 and CaRad53 to their human homolog Chk2 revealed that the C-terminal SCD is unique to the yeast proteins, while the N-terminal SCD is present in yeast Rad53 and human Chk2. Phosphorylation of the SCDs is closely associated with protein function and cell viability. For instance, phosphorylation at ScRad53-T354 and ScRad53-T358 in the activation loop is required for kinase activity [64]–[66]. Trans-phosphorylation of the ScRad53 N-terminal SCD is crucial for interaction with Dun1, the complex of which is involved in G2/M checkpoint [67]. Chk2-T68 phosphorylation is dependent on ATM/ATR and triggers Chk2 oligomerization, which led to PIKK-independent kinase activation [66], [68]. Furthermore, trans-phosphorylation at ScRad53-S485 and ScRad53-S489 by Mec1 and Tel1 kinases was shown to affect Rad53 oligomerization [63], [69]. Phosphorylation of this region is critical for protein function and hence cell viability [67], [69]. Thus, our finding of S461 in this region of CaRad53 as a potential site for phosphoregulation of cell's sensitivity to MMS may be explored as targets for developing for specific therapeutics to treat *C. albicans* infections.

## Materials and Methods

### Strains and culture conditions

All *C. albicans* strains used in this study are listed in Table 1. Except where noted. *C. albicans* were routinely grown at 30°C in YPD medium (2% yeast extract, 1% peptone, and 2% glucose), in GMM (2% glucose and 6.79 g/L yeast nitrogen base without amino acids), or in GMM supplemented with the required nutrients for auxotrophic mutants. Solid media contained 2% agar.

### Test of sensitivity to DNA damaging agents

Sensitivity to DNA-damaging agents was tested on solid or in liquid medium. For growth on solid media, cells were first grown in liquid YPD overnight at 30°C and 10-fold serially diluted with fresh YPD to concentrations of 1×10^2^ to 1×10^7^ cells/mL; after 2 h of growth at 30°C, 2 µL of each culture was plated onto YPD plates containing different concentrations of HU or MMS and the plates were photographed after 24 h incubation at 30°C. For liquid cultures, cells were grown in YPD at 30°C overnight and diluted with fresh YPD medium to a concentration of 5×10^6^ cells/mL. After 2 h incubation at 30°C, MMS or HU was added to a final concentration of 0.02% or 20 mM, respectively, and further incubated for 2–12 h before the cells were harvested.

For DNA damage recovery experiments, harvested cells were washed twice with distilled water after drug treatment, and resuspended in fresh YPD medium for further growth. For cell recovery rate assays, aliquots of 1×10^3^ cells/mL starting culture were collected at timed intervals after drug treatment and spread onto YPD plates where colony-forming units were counted after 1–2 d of incubation at 30°C.

### Construction of *C. albicans* mutant strains

*C. albicans* homologs of corresponding *S. cerevisiae* genes were identified in the *C. albicans* genome (http://www.candidagenome.org) by sequence alignment. *C. albicans* deletion mutants were constructed by sequentially deleting the two copies of the target gene(s) with two deletion cassettes from the wild-type strain of BWP17 [70], [71]. The two deletion cassettes were constructed by flanking a selectable marker gene (*ARG4* or *HIS1*) with the AB and CD DNA fragments (∼400 bp each), that correspond to the 5′ and 3′ untranslated regions (UTRs) of the target gene, respectively [21], [38]. Homozygous deletion mutants were verified by PCR.

For rescue experiments, the entire open reading frame (ORF) of the target gene, together with its promoter (−1,000 bp), was cloned into the CIp10-based, *URA3*-marked plasmid at KpnI and ClaI sites, followed by the GAL4 3′ UTR. The construct was linearized by StuI, whose site exists in the RP10 sequence of CIp10, and finally introduced into the gene deletion strains [21].

Construction of *PPH3* and *PSY2* deletion mutants carrying C-terminal Myc-tagged Rad53 was carried out as previously described [38].

To integrate C-terminal Myc-tagged Rfa2 into mutant strains of various mutation alleles of *RAD53*, we used the *URA3* flipper strategy described previously [72]. First, one copy of *RAD53* was replaced with *ARG4* as described above. Next, the coding sequence of the second copy of *RAD53* was deleted using a *URA3* flipper cassette, which was constructed by flanking the 4.2-kb *URA3* flipper with the AB and CD DNA fragments corresponding to the 5′ and 3′ UTR of target gene. 5-FOA was then applied to delete the *URA3* flipper cassette constructs. Then, different mutation alleles of *RAD53* were integrated using *HIS1* marker. Finally, C-terminal Myc-tagged Rfa2 cloned in CIp10-based, *URA3*-marked plasmid was linearized at a unique NsiI site, and transformed into different *RAD53* mutant strains described in the following context. Similar procedures were taken for double mutation.

Site-directed mutagenesis of *RAD53* was performed using the QuickChange Site-Directed Mutagenesis Kit (Stratagene, La Jolla, CA). Primers used in this study are listed in Table S1.

### Protein extraction, Western blotting and protein dephosphorylation

To extract proteins, cells were harvested by centrifugation, and ∼100 mg of cell pellet was resuspended in 300 µl of ice-cold RIPA buffer [16]. After adding an equal volume of acid-washed glass beads (Sigma-Aldrich), the cells were lysed by four rounds of 45 s of beating at 5,000 rpm in a MicroSmash MS-100 bead beater (Tomy Medico, Minato-ku, Japan) with 2 min of cooling on ice between rounds. Cell lysate supernatant was collected after centrifugation at 13,000 rpm for 20 min at 4°C. Protein concentration of the lysate was determined using bicinchoninic acid (BCA) protein assay (Galen).

For Western blotting, 30 µg of total protein was separated by 10% or 12% SDS-PAGE and transferred to a polyvinylidene difluoride (PVDF) membrane (Millipore). The membrane was immersed in Tris-buffered saline containing 0.1% Tween 20 (TBST) and 5% non-fat dry milk for 1 h at room temperature, followed by primary antibody and secondary antibody conjugated with hydrogen peroxidase (HRP) or alkaline phosphatase (AP) consecutively for 1 h each, both in TBST containing 1% milk. The target protein was visualized by using an enhanced-chemiluminescence (ECL) system or Alkaline Phosphatase (AP) system. Anti-Myc and anti-Cdc28 (PSTAIRE) antibodies were puchased from Santa Cruz (USA).

Protein dephosphorylation was carried out as described previously [30]. Lambda phosphatase was purchased from New England BioLabs (catalog no. P07535).

### Microscopy and flow cytometry

Staining of nuclei and chitin was carried out as previously described [5]. Zeiss 510 metamicroscope and Cell Observer system (Carl Zeiss MicroImaging, Germany) were used for imaging. Flow cytometry was performed on Flow Cytometer BD FACSVantage™ SE system as described previously [21].

### RNA isolation, RT-PCR, Northern blot and qPCR

Total RNA was obtained as previously described [73]. cDNA was synthesized using the SuperScript II Reverse Transcriptase kit (Invitrogen). RT-PCR was done according to the description of Kelly, *et al*. (2004) [74]. Northern blot was performed according to the description of Lane, *et al*. (2001) [75]. qPCR was performed using the iQ SYBR Green Supermix (Bio-Rad) and detected via the iCycler iQ detection system (Bio-Rad). Oligonucleotide primers used to detect the transcripts of selected genes by RT-PCR are shown in Table S1. qPCR was done using the following program: initial denaturation at 94°C for 5 min, followed by 40 cycles of 94°C for 20 s, 56°C for 30 s, and 68°C for 20 s. Amplification specificity was determined by melting curve analysis.

## Supporting Information

Table S1 **Oligonucleotide primers used for Construct, RT-PCR and qPCR analysis.**
(DOC)

Figure S1 ***pph3Δ*** **and** ***psy2Δ*** **cells exhibit pseudohyphal growth upon MMS treatment.** Fig. S1. Wild-type (SC5314 or BWP17), *pph3Δ* (SJL3) and *psy2Δ* (SJL6) cells were grown in liquid YPD medium supplemented with 0.02% MMS at 30°C for 6 h, washed with fresh YPD and resuspended into fresh YPD for further growth at 30°C for 8 h. Cells were collected for microscopic examination at the indicated times. (Bar = 5 µm).
(TIF)

Figure S2 ***pph3Δ*** **and** ***psy2Δ*** **cells exhibit pseudohyphal growth upon HU treatment.** Fig. S2. Wild-type (SC5314 or BWP17), *pph3Δ* (SJL3) and *psy2Δ* (SJL6) cells were grown in liquid YPD medium supplemented with 20 mM HU at 30°C for 6 h, washed with fresh YPD and resuspended into fresh YPD for further growth at 30°C for 8 h. Cells were collected for microscopic examination at the indicated times. (Bar = 5 µm).
(TIF)

Figure S3 **Rad53p undergoes hyperphosphorylation in response to HU and MMS.** Fig. S3A Rad53 hyperphosphorylation in HU-treated cells. The lysate of SJL9 (wild-type with *RAD53-Myc*), SJL7 (*pph3Δ RAD53-Myc*), and SJL8 (*psy2Δ RAD53-Myc*) cells that were incubated at 30°C in YPD containing 20 mM HU for 4 h was divided into 2 parts. One was treated with λ-phosphatase (PPase), and the other was mock-treated with the reaction buffer alone. The two samples, along with untreated cell lysates, were then subjected to Western blot analysis using anti-Myc antibody. Fig. S3B Rad53 hyperphosphorylation in MMS-treated cells. The lysate of SJL9 (wild-type with *RAD53-Myc*), SJL7 (*pph3Δ RAD53-Myc*), and SJL8 (*psy2Δ RAD53-Myc*) cells that were incubated at 30°C in YPD containing 0.02% MMS for 4 h was divided into 2 parts. One was treated with λ-phosphatase (PPase), and the other was mock-treated with the reaction buffer alone. The two samples, along with untreated cell lysates, were then subjected to Western blot analysis using anti-Myc antibody.
(TIF)

Figure S4 **Detection of Rad53 hyperphosphorylation by Western blotting in** ***pph3Δ*** **cells and cells expressing various mutant alleles of** ***RAD53*** **upon HU treatment.** Fig. S4. Wild-type (SJL9), *pph3Δ* (SJL7), and the various strains expressing C-terminally Myc-tagged mutant alleles of *RAD53* (HT13.1–16.1 *rad53-S461A*, *rad53-S461D*, *rad53-S545A*, *rad53-S545D* cells were incubated at 30°C in YPD containing 20 mM HU for 4 h, then washed and recovered with fresh YPD for the indicated times. Whole cell lysates were used for immunoblot analysis with anti-Myc antibody. Untreated cells were used as control. Cdc28 was probed with anti-PSTAIRE antibody as loading control.
(TIF)
