# Supplementary material for: Pph3 Dephosphorylation of Rad53 Is Required for Cell Recovery from MMS-Induced DNA Damage in Candida albicans
Source: PLoS One. 2012 May 14;7(5):e37246. doi: 10.1371/journal.pone.0037246 (PMC3351423; doi:10.1371/journal.pone.0037246)
Supplement: Table S1 — Oligonucleotide primers used for Construct, RT-PCR and qPCR analysis. (DOC) [file pone.0037246.s001.doc]

**Table S1.** Oligonucleotide primers used for Construct,RT-PCR and qPCR analysis.

|  | **Primers** | **Sequence^a^, 5'-3'** |
| --- | --- | --- |
| WB | RFA2-myc F | ACTGGTACCATGTCAGATTTTGAATACGATAATAAC |
|  | RFA2-myc R | TAACTCGAGAATCACAAGATAACTGTTGTCATC |
| Knock Out | RAD53 URA3-flipper A | CGGGGTACCCCGTTGGTGCTGCCATTGGTATTG |
|  | RAD53 URA3-flipper B | CCGCTCGAGAAGATCAAGATGAAAACTAGAGACC |
|  | RAD53 URA3-flipper C | AAGGAAAAAAGCGGCCGCCTAGCTCTTGGTCGTTGAGATTCCAC |
|  | RAD53 URA3-flipper D | ATACCGCGGGCTTGATATTTGAGGAACTCACTCCTG |
| Mutantion | Rad53 S327A F | CAGTGACAATCTGGCTTTTATGAAAAC |
|  | Rad53 S327A R | GTTTTCATAAAAGCCAGATTGTCACTG |
|  | Rad53 S327D F | CAGTGACAATCTGGATTTTATGAAAAC |
|  | Rad53 S327D R | GTTTTCATAAAATCCAGATTGTCACTG |
|  | Rad53 S350A F | CGGTAAGTATGGAGCTTCGCAGATGGAAC |
|  | Rad53 S350A R | GTTCCATCTGCGAAGCTCCATACTTACCG |
|  | Rad53 S350D F | CGGTAAGTATGGAGATTCGCAGATGGAAC |
|  | Rad53 S350D R | GTTCCATCTGCGAATCTCCATACTTACCG |
|  | Rad53 S351A F | GTAAGTATGGATCAGCTCAGATGGAACTGC |
|  | Rad53 S351A R | GCAGTTCCATCTGAGCTGATCCATACTTACCG |
|  | Rad53 S351D F | GTAAGTATGGATCAGATCAGATGGAACTGC |
|  | Rad53 S351D R | GCAGTTCCATCTGATCTGATCCATACTTACCG |
|  | Rad53 S461A F | GAGTTTATCGCAATCACAGGCTCAACAATCTCG |
|  | Rad53 S461A R | TTCGAGATTGTTGAGCCTGTGATTGCGATAAAC |
|  | Rad53 S461D F | GAGTTTATCGCAATCACAGGATCAACAATCTCG |
|  | Rad53 S461D R | TTCGAGATTGTTGATCCTGTGATTGCGATAAAC |
|  | Rad53 S545A F | GAACGCTTGACAAGCAAGAAAGTCAAGC |
|  | Rad53 S545A R | TGACTTTCTTGCTTGTAGCAGGTTCTTCC |
|  | Rad53 S545D F | GAACGATTGACAAGCAAGAAAGTCAAGC |
|  | Rad53 S545D R | TGACTTTCTTGCTTGTATCAGGTTCTTCC |
|  | Rad53 S695A F | CTTATTTCGGGTTTAGCTAGTATAAGTTC |
|  | Rad53 S695A R | TGAACTTATACTAGCTAAACCCGAAATAAG |
|  | Rad53 S695D F | CTTATTTCGGGTTTAGATAGTATAAGTTC |
|  | Rad53 S695D R | TGAACTTATACTATCTAAACCCGAAATAAG |
|  | Rad53 S4A F | GGCTTTAGCTCAAGCTCAGGCTCAACAATCTCG |
|  | Rad53 S4A D | TTGAGCCTGAGCTTGAGCTAAAGCCAATGATTTGAC |
|  | Rad53 S4D F | GGATTTAGATCAAGATCAGGATCAACAATCTCG |
|  | Rad53 S4D D | TTGATCCTGATCTTGATCTAAATCCAATGATTTGAC |
| RT-PCR | CCN1 F | CTTTACAACAACAAAGAGTCAAATATGG |
|  | CCN1 R | AGACATAAAAATAATGTTGTTGGTTGTAAT |
|  | PCL2 F | AAATATTCTCAAGACAGCCAGTAT |
|  | PCL2 R | GATGTCTAGTAGTTTCCATCCCTAT |
|  | RFA2 F | TGAATACGATAATAACAATACTGGA |
|  | RFA2 R | CATTTACGAACATCAATAGACC |
|  | MSH2 F | AGTACCAAAATAGATCTCAAATTCA |
|  | MSH2 R | TTTGCTATGATAAACTTCGACTT |
|  | MSH6 F | TATCAACTCCTACAAGATCATCAC |
|  | MSH6 R | CTAATTGTGGTTTGATTTGTTTATC |
|  | CLB4 F | GAAAATGAGTTGACAAAACAAAG |
|  | CLB4 R | TCATTTAGTAATACCTTCTCCAATC |
|  | SIC1 F | TCTTCTAATGATACACCATCTTTATT |
|  | SIC1 R | TTTTTCGTTACTAGTGAATAAATCC |
|  | HGC1 F | TAACTAAACCATTAACTCCAAAATC |
|  | HGC1 R | TAATCGAATGGTGAAAGTTAAATA |
|  | EBP1 F | ATTTGTTGTCCCATCAGATACTA |
|  | EBP1 R | ACTTTTAGCTTGAGCGTCATTAT |
|  | ECE1 F | GAAATTCTCCAAAATTGCCTGT |
|  | ECE1 R | AACAACAGAATCAATATCTTCTCTC |
|  | HWP1 F | ACTGCTCAACTTATTGCTATCGCTT |
|  | HWP1 R | TTCTTGTGGTTGTTGTGGGTAGTC |
|  | ALS3 F | TGTTACTCATATATTTGTCGGTTG |
|  | ALS3 R | ATAAATTCTTCACCTGCCTGAAAT |
|  | SAP4 F | TGTTCTTACAAAATATCTTGAGTGT |
|  | SAP4 R | TATTTGAACCAATCGTAATATCAG |
|  | RBT1 F | GCCCTCGCTTACTACATTTTAT |
|  | RBT1 R | AGTTACAATCAATACCATAAGAACC |
|  | UME6 F | ATGATTACCCATATGGTTACACCCG |
|  | UME6 R | CATTGGTTATATCATTACTTGATTTTTTCC |
|  | GAPDH F | ATTGGTATTAACGGTTTCGGTAGAATCGGTA |
|  | GAPDH R | AAAACACCGGTGGATTCAATAACGTAGTCA |
| qPCR | qCCN1 F | GATGGTAGTGGCAGTGATGG |
|  | qCCN1 R | ATATTGTGAAGTTGCTGAAGGTG |
|  | qMSH2 F | CAAGGACAACGAGCAGGATA |
|  | qMSH2 R | CACCGTTCAACAAATCCAAG |
|  | qPCL2 F | ACCCTTTCTTGCAACCTATCA |
|  | qPCL2 R | TTCTGAAGAACTAGAAGAATGTAAGGA |
|  | qRFA2 F | CCAGTGCTGGTAGTGGATTG |
|  | qRFA2 R | GTCAACTGGGACACCATCTG |
|  | qGAPDH F | GTCAACGATCCATTCATTGC |
|  | qGAPDH R | GACCATCAATGACCAAGTCG |
|  | qHWP1 F | ATCCCAGATATTCCAGAAAAGCCAA |
|  | qHWP1 R | TTTGGAGTGGTAGCTGGAACAGAAG |
| Northern | nHWP1 F | TAATCATCCTCAATCAATGGTTCCG |
|  | nHWP1 R | TGATTGGGTCACTTGTTGTTGTTGT |
|  | nPCL2 F | CAAACCACTGAAGAGAAACCAGCAT |
|  | nPCL2 R | AGAACCAACGGCTTTAGATTTTTGC |
|  | nRFA2 F | ATTAATGATGCAACACAACCAGTGC |
|  | nRFA2 R | TCAATACGGCTATCACTTGATCCGT |
|  | nCCN1 F | GATGAGAACCAACCACCACAAATTC |
|  | nCCN1 R | CGGAACCATTGATTGAGGATGATTA |
|  | nMSH2 F | GGTACCGCAGGAAACTTGGATAGTC |
|  | nMSH2 R | TTCCATTTGGTAAAATGTTCAACGC |
|  | nGAPDH F | AAGAGTTGCTTTGGGCAGAAAAGAC |
|  | nGAPDH R | TTCTACCACCTCTCCAGTCCTTGTG |

a. Added restriction sites are underlined.
