# Supplementary figures and images for: Pph3 Dephosphorylation of Rad53 Is Required for Cell Recovery from MMS-Induced DNA Damage in Candida albicans
Source: PLoS One. 2012 May 14;7(5):e37246. doi: 10.1371/journal.pone.0037246 (PMC3351423; doi:10.1371/journal.pone.0037246)

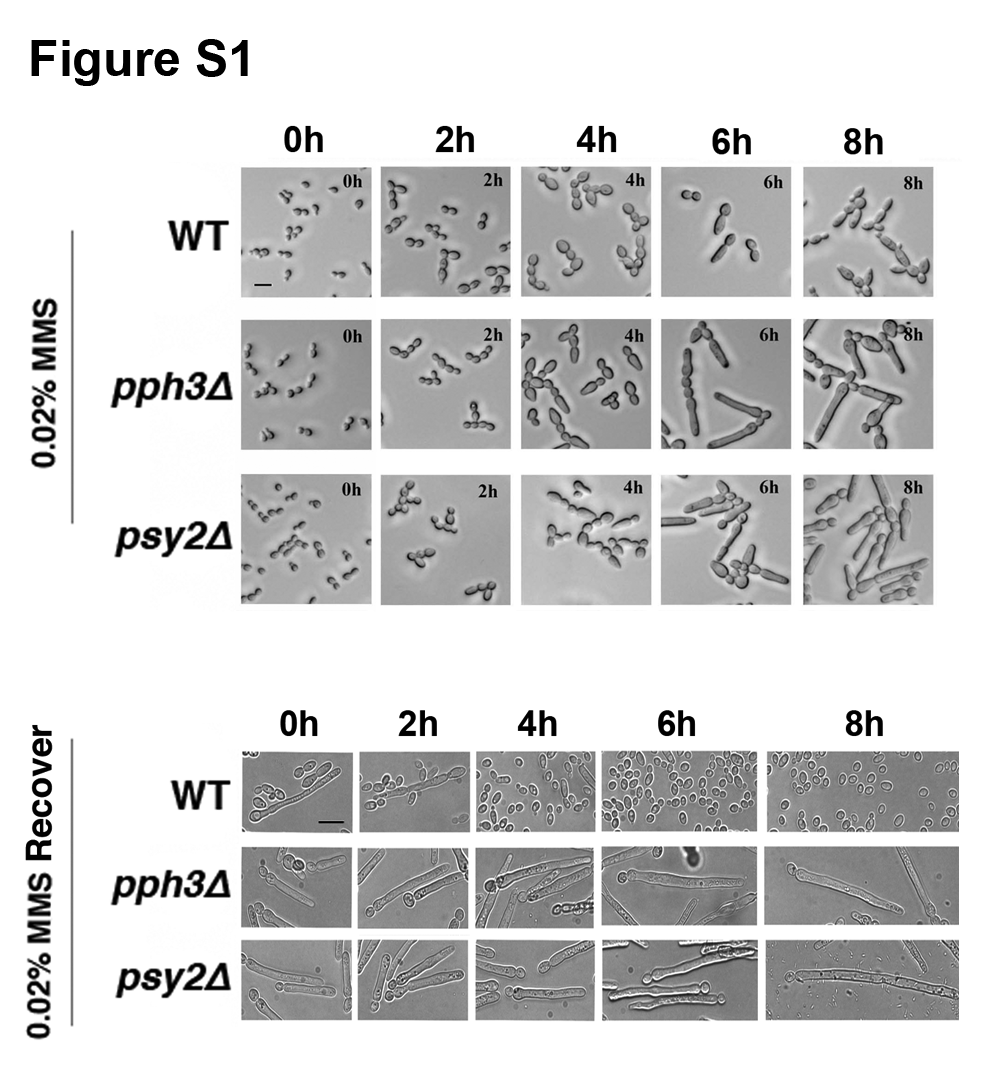

Supplement: Figure S1 — pph3Δ and psy2Δ cells exhibit pseudohyphal growth upon MMS treatment. Fig. S1. Wild-type (SC5314 or BWP17), pph3Δ (SJL3) and psy2Δ (SJL6) cells were grown in liquid YPD medium supplemented with 0.02% MMS at 30°C for 6 h, washed with fresh YPD and resuspended into fresh YPD for further growth at 30°C for 8 h. Cells were collected for microscopic examination at the indicated times. (Bar = 5 µm). (TIF) [file pone.0037246.s002.tif]

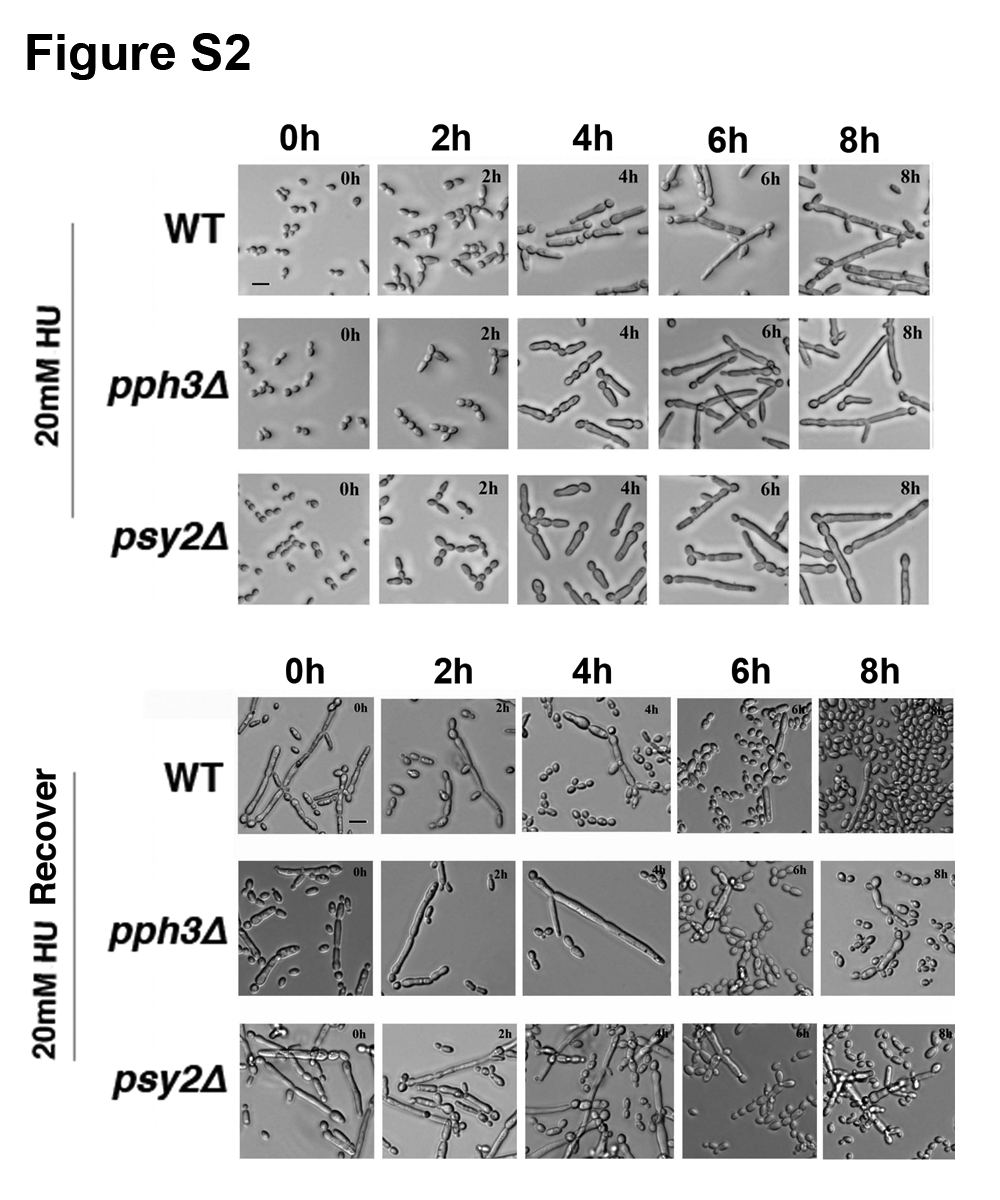

Supplement: Figure S2 — pph3Δ and psy2Δ cells exhibit pseudohyphal growth upon HU treatment. Fig. S2. Wild-type (SC5314 or BWP17), pph3Δ (SJL3) and psy2Δ (SJL6) cells were grown in liquid YPD medium supplemented with 20 mM HU at 30°C for 6 h, washed with fresh YPD and resuspended into fresh YPD for further growth at 30°C for 8 h. Cells were collected for microscopic examination at the indicated times. (Bar = 5 µm). (TIF) [file pone.0037246.s003.tif]

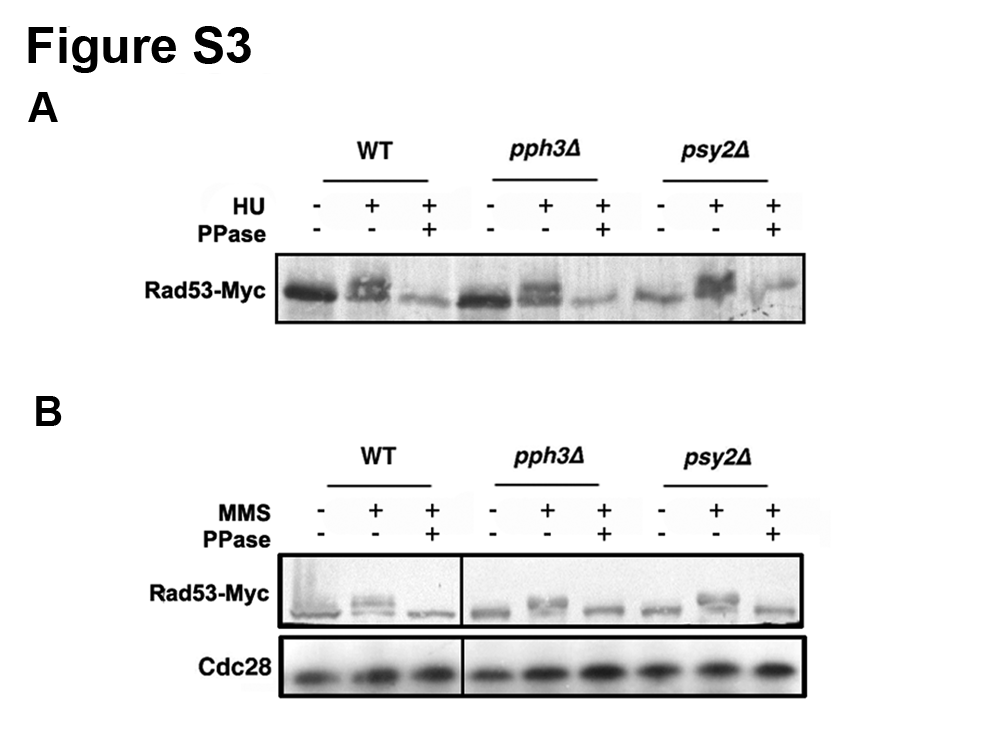

Supplement: Figure S3 — Rad53p undergoes hyperphosphorylation in response to HU and MMS. Fig. S3A Rad53 hyperphosphorylation in HU-treated cells. The lysate of SJL9 (wild-type with RAD53-Myc), SJL7 (pph3Δ RAD53-Myc), and SJL8 (psy2Δ RAD53-Myc) cells that were incubated at 30°C in YPD containing 20 mM HU for 4 h was divided into 2 parts. One was treated with λ-phosphatase (PPase), and the other was mock-treated with the reaction buffer alone. The two samples, along with untreated cell lysates, were then subjected to Western blot analysis using anti-Myc antibody. Fig. S3B Rad53 hyperphosphorylation in MMS-treated cells. The lysate of SJL9 (wild-type with RAD53-Myc), SJL7 (pph3Δ RAD53-Myc), and SJL8 (psy2Δ RAD53-Myc) cells that were incubated at 30°C in YPD containing 0.02% MMS for 4 h was divided into 2 parts. One was treated with λ-phosphatase (PPase), and the other was mock-treated with the reaction buffer alone. The two samples, along with untreated cell lysates, were then subjected to Western blot analysis using anti-Myc antibody. (TIF) [file pone.0037246.s004.tif]

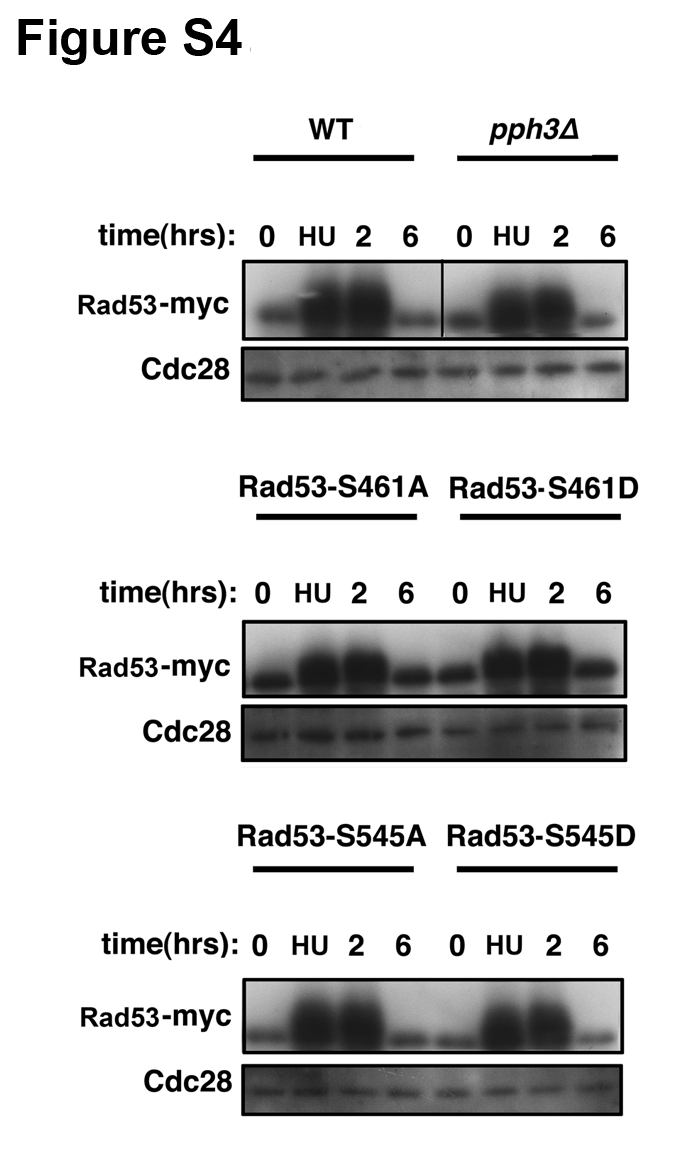

Supplement: Figure S4 — Detection of Rad53 hyperphosphorylation by Western blotting in pph3Δ cells and cells expressing various mutant alleles of RAD53 upon HU treatment. Fig. S4. Wild-type (SJL9), pph3Δ (SJL7), and the various strains expressing C-terminally Myc-tagged mutant alleles of RAD53 (HT13.1–16.1 rad53-S461A, rad53-S461D, rad53-S545A, rad53-S545D cells were incubated at 30°C in YPD containing 20 mM HU for 4 h, then washed and recovered with fresh YPD for the indicated times. Whole cell lysates were used for immunoblot analysis with anti-Myc antibody. Untreated cells were used as control. Cdc28 was probed with anti-PSTAIRE antibody as loading control. (TIF) [file pone.0037246.s005.tif]
